# Design and characterization of an acoustic composite lens with high-intensity and directionally controllable focusing

**DOI:** 10.1038/s41598-020-58092-6

**Published:** 2020-01-30

**Authors:** Hongyu Sun, Shen Wang, Songling Huang, Lisha Peng, Qing Wang, Wei Zhao

**Affiliations:** 1 State Key Laboratory of Power System, Department of Electrical Engineering, Tsinghua University, Beijing, 100084 China; 2 Department of Engineering, Durham University, Durham, UK

**Keywords:** Composites, Acoustics

## Abstract

Acoustic orientation and bunching methods, which include the radiation surface expansion, ultrasonic demodulation, multiunit coherence, phased arrays and acoustic lenses, can be used to manipulate and focus sound waves. Recently, focusing systems composed of acoustic lenses have been found to offer high controllability and focusing intensity. In this paper, a newly designed composite acoustic lens that can achieve wave convergence is proposed by assembling a lattice array of concave hexagonal (CH)-shaped rods. In comparison with the latest published work, the new CH structure improves upon the focusing capability of traditional acoustic lenses while retaining their advantages in terms of 3-D underwater focusing. Simulated and experimental results show that a lens with the CH structure has good focusing intensity and can focus acoustic waves over a wide range of incidence angles without losing its functionality. With its good focusing capabilities, this new composite lens may open the door to a broad range of applications, including high-precision nondestructive testing (NDT), high-efficiency medical treatment and multidirectional underwater focusing.

## Introduction

Over the past three decades, man-made materials that can control wave characteristics have been proposed and endowed with capabilities beyond those of materials that exist in nature^1–4^. Periodic composites that act as special unnatural structures such as photonic^5^ or phononic crystal arrays have been theoretically developed and experimentally verified^6,7^. In contrast to metamaterial-based negative refractive index devices with deep-subwavelength resolution^8–13^, periodic crystal structures introduce acoustic waves into phononic crystals resulting from Bragg scattering and occurring in passbands with a negative group velocity^14,15^. However, in practice, for usage in medical treatment and nondestructive testing (NDT), high-performance acoustic composite materials are required, such as acoustic superlens or hyperlens^16^. To overcome the limitations of such materials for these potential applications, the focusing of acoustic waves using phononic crystals has been systematically studied in both air and water^17,18^, and a broad variety of applications for acoustic focusing have been demonstrated.

In the literature, in the field of acoustic focusing with composite lens structures, resonant units for convergent lenses have been designed and developed with various shapes, such as rigid cylinders^19–22^, Helmholtz resonators^23,24^, cross structures^25–27^, and concentric rings^28^, or with the use of multiphase materials to reduce impedance mismatch^29^. Acoustic lenses with rigid cylinders are commonly designed as gradient index (GRIN) homogenized 2-D sonic crystals based on Bragg reflection^30^. To modify the local refraction index (or filling fraction) to achieve sound focusing, a particular calculated radial distribution or crystal material must be used for the cylinders, as seen from both theory and experiment^31^. However, 2-D GRIN acoustic lenses with flat surfaces cannot achieve 3-D focusing, and difficulties arise in manufacturing them with specific sizes or material property distributions. Asymmetrical Helmholtz resonators^17^, which provide better impedance matching and a higher refractive index, are also difficult to process, although they can achieve a better acoustic focusing effect. Furthermore, unit cells with the “ + ”- or cross-shaped (CS) structure have a high effective density because of the small gaps minimizing the total volume fraction^32^, thus allowing a perfect impedance match to be established between the lens and water. A recent study has shown both numerically and experimentally that 3-D underwater focusing can be achieved using CS-structured single-phase units of small sizes due to the anisotropic dispersion in the first band^27^. To the best of our knowledge, ref. ^27^ reported the latest and most effective method for achieving acoustic wave focusing with the CS structure^25–27^, in which underwater acoustic wave focusing is achieved in a three-dimensional manner, offering the best focusing capability to date.

In this work, we develop a composite acoustic lens with an improved focusing capability by changing the shape of the unit cell from the traditional CS structure to the new concave hexagonal (CH) structure, which enables smaller gaps and a smaller volume fraction. Moreover, we find that this new composite lens can achieve multidirectional focusing without losing its wave focusing intensity. We compare the two structures through simulations and experiments and find that the characteristics of the CS structure are nearly consistent with those of the “ + ”-shaped rods in refs. ^25–27^, thus proving the effectiveness of the method used in this study. The results show that the newly designed CH unit cell structure for composite lenses improves the 3-D focusing intensity for underwater acoustic waves and enables multidirectional focusing over a wide range of bias angles.

## Methods

**Figure 1:**
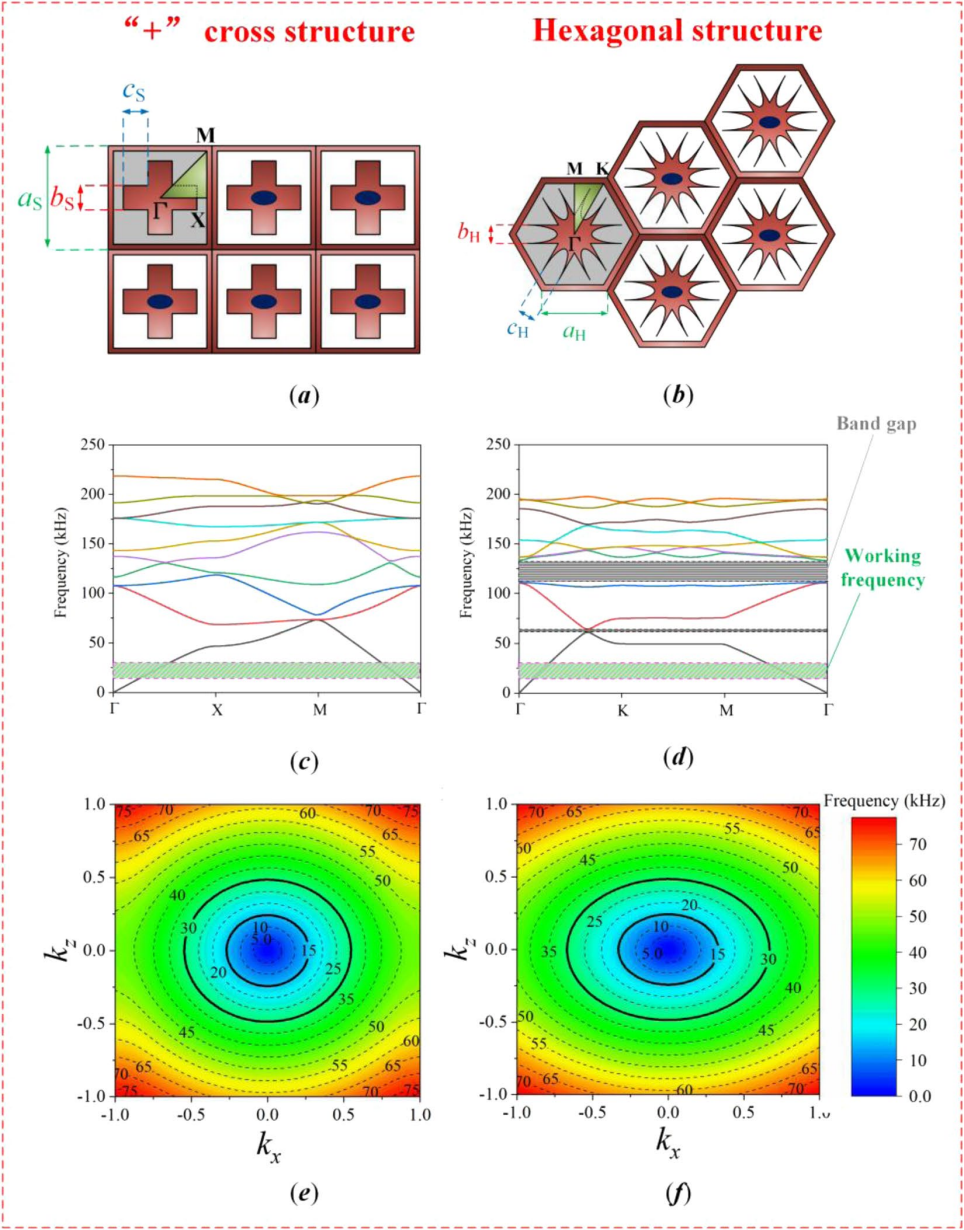
Structural layouts and other properties of the CS and CH structures: (**a**,**b**) The shape of each unit cell. *a*_S_ and *a*_H_ are the lattice constants, *b*_S_ and *b*_H_ are the widths, and *c*_S_ and *c*_H_ are the lengths. The irreducible Brillouin zone is Γ-X-M-Γ in (**a**) and Γ-K-M-Γ in (**b**). (**c**,**d**) Band structures in the *x*-*y* plane. The green strips indicate the range of working frequencies (15-30 kHz), and the gray strips represent band gaps. (**e**,**f**) EFCs in the *x*-*z* plane, where the shape for the CH structure is flatter than that for the CS structure.

The theory and design method applied in this work are inspired by the CS-structured lenses proposed in recent studies^25–27^, and we compare the performance of two different structures in this paper. Specifically, the characteristics of acoustic lens with the CS and CH structures are investigated in this paper, as presented in Fig. 1. Unit cells with these two structures are depicted in Fig. 1(a,b), respectively, and the first cell in each image is annotated to show more detail. The lattice parameters and working frequency are listed in Table 1.

**Table 1:** Simulation parameters.

Parameter		Value
Frequency (kHz)		19
Lattice constant (mm)	*a*_S_	12
	*a*_H_	6
Width (mm)	*b*_S_	2
	*b*_H_	2
Length (mm)	*c*_S_	2
	*c*_H_	2

**Figure 2:**
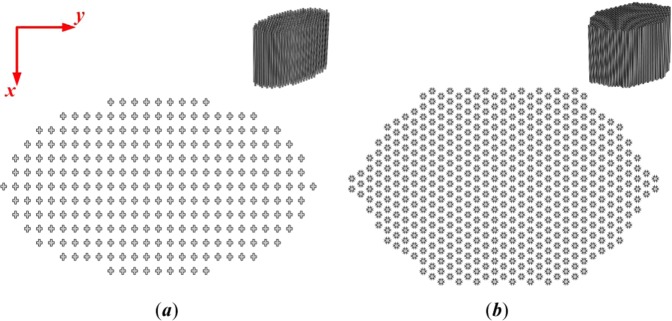
Structural layouts with the two different unit cells: (**a**) CS structure. (**b**) CH structure.

The irreducible Brillouin zones in the *x*-*y* plane are defined in Fig. 1(a,b) to obtain the dispersion relationships of the two structures. To calculate the band structures (Fig. 1(c,d)), eigenvalue analysis was performed in COMSOL using the finite element method (FEM) in accordance with the Floquet-Bloch theorem^33^. Working frequencies (marked in green) of 15-30 kHz are available for both structures, although there are also two band gaps (marked in gray) for the CH structure. Accordingly, a working frequency of 19 kHz is suitable for both structures considered in this work. Moreover, to enable 3-D focusing with each composite lens, the equifrequency contours (EFCs) in the *x*-*z* plane were computed by sweeping the wavevector through all positions in the unit cell^34–36^. As shown in Fig. 1(e, f), the EFCs of the two structures are both elliptical and anisotropic but have different shapes, which determine the wave propagation directions and focusing mechanisms in the *x*-*z* plane. Moreover, Fig. 2(a, b) show the structural layouts of the two types of acoustic lenses, where a biconvex periodic array of units is used to achieve wave focusing^37^. The material parameters are listed in Table 2.

**Table 2:** Material parameters.

Parameter	Value
Density of water (kg/m^3^)	1000
Sound speed (m/s)	1475
Temperature (K)	293.15
PML reference speed (m/s)	1475

To study the focusing capability of each structure, FEM-based numerical simulations were performed to calculate the sound pressure distribution, which has been verified through theory and experiments in previous studies^25,26^. The governing equations used in the simulations included the momentum and mass conservation equations, as follows^38^:

1 $$j\omega {\boldsymbol{\rho }}({\boldsymbol{r}}){\rho }_{0}{\boldsymbol{v}}=-\nabla p,$$

2 $$j\omega p=-B({\boldsymbol{r}}){B}_{0}\nabla \cdot v{\boldsymbol{,}}$$

where *p* is the wave pressure, ***v*** is the velocity vector, *B*(***r***) is the relative bulk modulus and ***ρ*** is the relative density tensor. For simplicity, the parameters of water were used for *B*_0_ and *ρ*_0_. The incident wave was defined as a plane wave propagating along the *x*-direction in the simulations. In the experimental configuration, a point source was used to generate spherical waves and placed 200 mm away from the composite lens. Spherical waves can be approximated as plane waves when *kr*≫1, thus demonstrating the rationality of this experimental approximation^27^. To avoid wave reflection from the boundaries, perfectly matched layer (PML) conditions were utilized at the boundaries of the model.

## Results and Discussion

**Figure 3:**
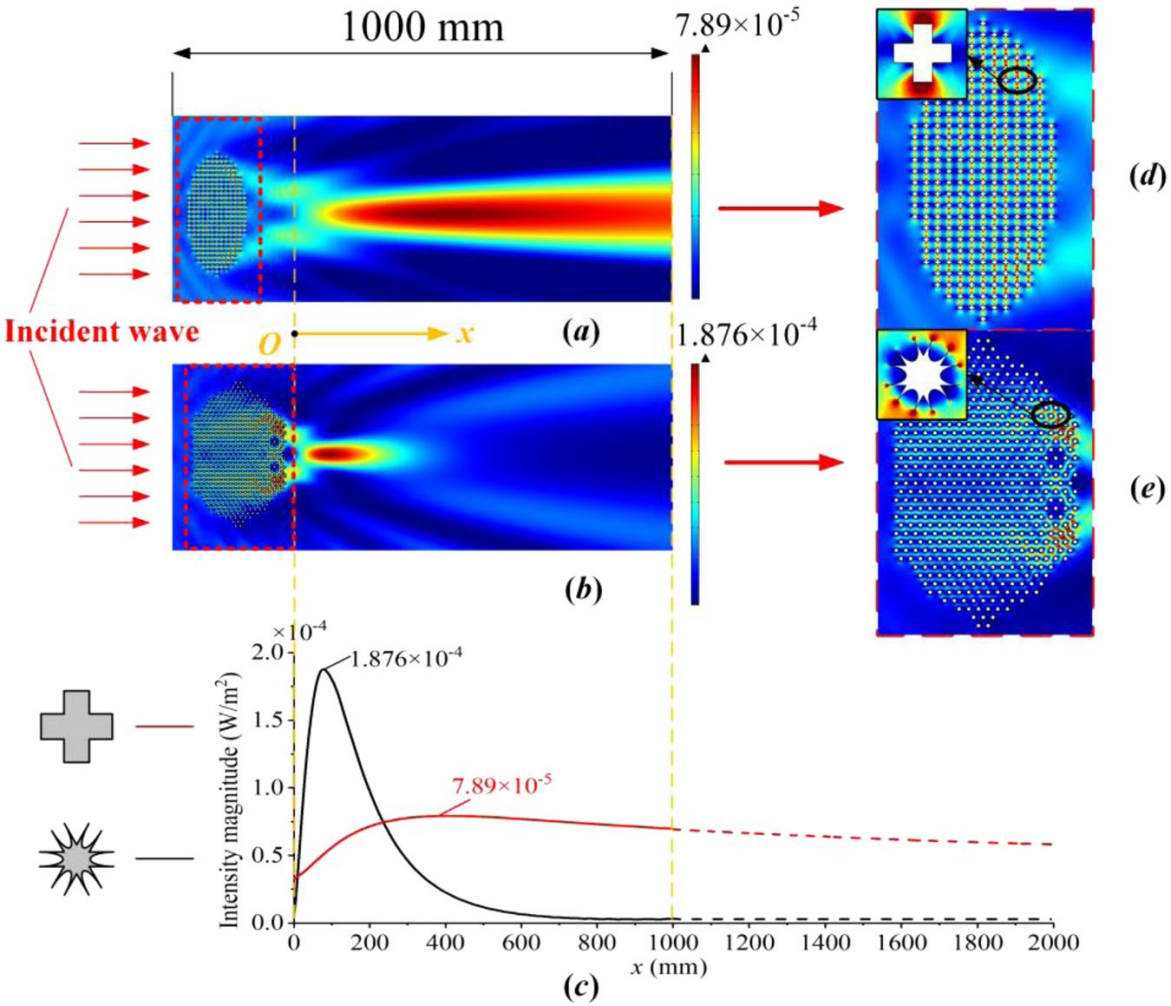
Simulation results for acoustic lenses with different structures: (**a**,**b**) Wave intensity field distributions under parallel incident waves, where the total calculated length in the *x*-direction in the simulation is 1000 mm. (**c**) Intensity distribution along the *x*-axis at the center of each lens. The origin of the coordinate system is selected to be the position before the two focal points, and the two structures share the same origin and terminal positions. To describe the subsequent trends of the wave intensity distribution along the *x*-direction, black and red dashed lines are plotted to represent the additional simulation data. (**d**,**e**) Partial magnified views of the differently structured lenses in (**a**,**b**), as indicated by the dotted red rectangles, and the sound field for each unit cell.

**Figure 4:**
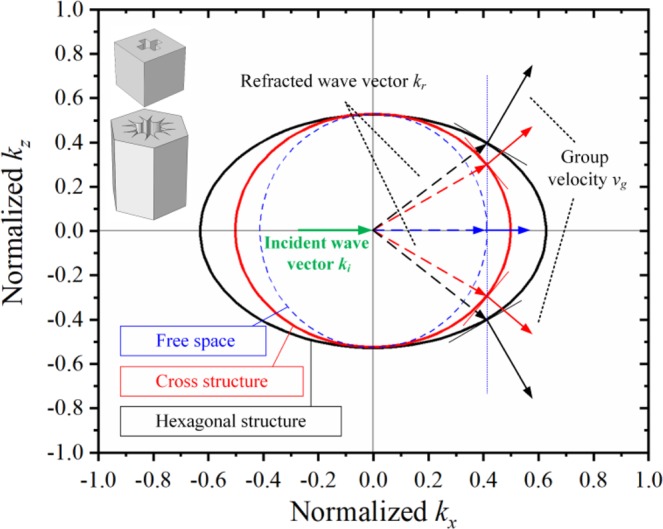
EFCs for the CH structure, the “ + ”-shaped (CS) structure and free space. The wave vectors *k*_*x*_ and *k*_*y*_ are normalized, and the cell models are shown here. The three EFCs have identical incident wave vectors *k*_*i*_ (green solid arrow), but the refracted wave vectors *k*_*r*_ (dotted arrows) vary. Because the wave directions must be normal to the EFCs, the directions of the group velocities (solid arrows) also vary.

**Figure 5:**
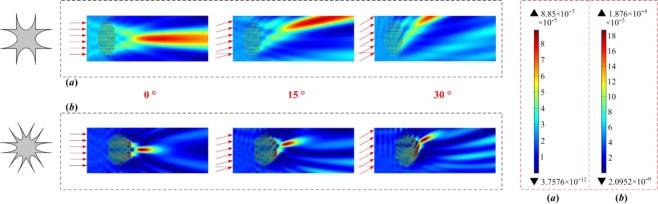
Wave intensity distributions for each cell shape at different incidence angles; here, the CS structure is changed to the concave “ + ”-shaped structure to minimize the structural differences between the two lenses.

Figures 3–5 show the simulation results for the different structures at a frequency of 19 kHz. The wave intensity distributions for the CS and CH structures are presented in Fig. 3(a, b), and the intensity distributions along the centerline in the *x*-direction are shown in Fig. 3(c). The CH-structured composite lens shows evident focusing of the waves with an intensity of 1.876 × 10^-4^ W/m^2^, more than twice as large as that of the CS-structured lens, which has a focusing intensity of 7.89 × 10^-5^ W/m^2^. Moreover, the focal length of the CS-structured acoustic lens is nearly four times longer than that of the CH-structured lens. Partial magnified view of the acoustic lenses, enclosed by dotted red rectangles, show the wave bending phenomenon and the pressure on the unit cells (see Fig. 3(d, e)), and the focusing path is clearly shown for each structure. In addition, for illustration, the EFCs for the two structures (ellipses) and for free space (circle) have been determined and plotted in Fig. 4^16^. The group velocity ***v***_*g*_ is defined as^36^

3 $${{\boldsymbol{v}}}_{g}={\nabla }_{k}\omega ,$$

and the propagation directions of the waves are normal to the EFCs^16,39^. Furthermore, each composite lens can be treated as a homogeneous materials represented by effective material parameters because of the limited size of the unit cell (the wave-length is *λ* = *c*/*f* = 77.6 mm when the working frequency is 19 kHz, while the lattice constant is 12 mm)^25^. Because the pressure wave mainly propagates through areas of higher density, reducing the bulk modulus minimizes the impedance mismatch between the water and the lens material. Accordingly, for the CH-structured composite lens, its high effective density *ρ*^*x*^_eff_ and its a low effective bulk modulus *B*_eff_ are beneficial for energy convergence and loss reduction. Therefore, the acoustic lens with the CH structure has a better focusing capability than either the CS-structured lens or free space does.

For incident waves with different angles of inclination relative to the *x*-axis, the performances of the acoustic lenses with the CS and CH structures were compared through numerical simulations. As shown in Fig. 5(a, b), the focusing capability of the lens with the concave cross-shape (CCS) structure (the CS structure was changed to the concave “ + ”-shaped structure to minimize the structural differences between the two lenses) decreases with an increase in the inclination angle, while the acoustic lens with the CH structure has a stable focusing intensity and a movable focal position as when the angle changes. Therefore, the newly designed CH-structured lens can achieve multidirectional focusing without loss of performance.

**Figure 6:**
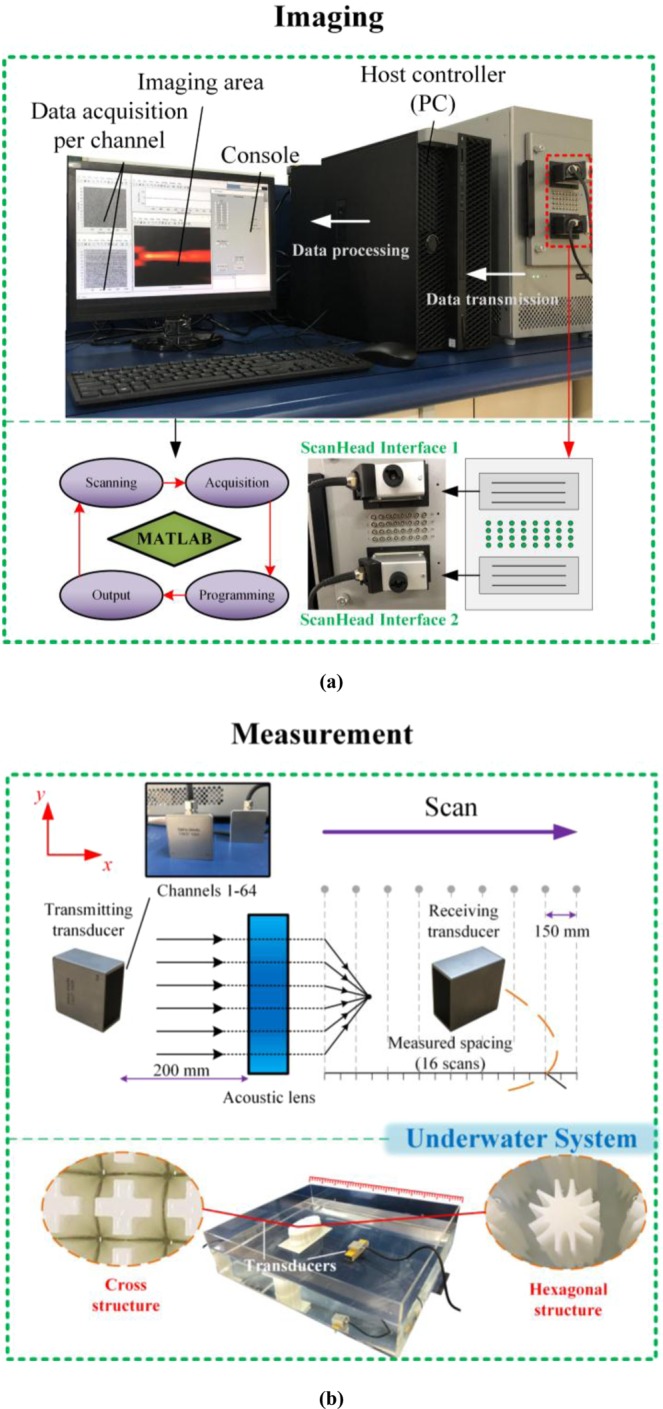
Experimental configuration of the 3-D underwater ultrasonic focusing system. The composite lenses with different unit cell shapes were manufactured using 3-D printing technology. The size of the acrylic water tank was 800 × 500 × 150 mm^3^_,_ whereas the height of the acoustic lenses was 100 mm in this work.

To verify the theoretical predictions and simulation results, experiments were set up to measure the wave intensities of acoustic composite lenses with the different cell structures (see Fig. 6). The experimental configuration included systems for imaging and measurement. For the measurement system, a 64-channel transmitting transducer was placed 200 mm from the composite lens and could transmit ultrasonic waves in any direction over a wide range of frequencies (only the center channel was used to act as a point source), and another transmitting transducer (also referred to as the receiving transducer) could receive ultrasonic waves at all positions. To measure the acoustic field at different points, 16 scans were performed along the *x*-direction with 75 mm gaps, and the data received in each channel were transmitted to the host controller. For the probes in this experiment, water was selected as the officially specified impedance-matched medium; therefore, the best measurement results could be obtained in an underwater system in this work. For the imaging system, the received ultrasound signals were amplified as a function of the time from the transmission event by the computer; then, they were digitized by 14-bit analog-to-digital converters at an appropriate sampling rate. By processing the acquired data, we could display the image of the acoustic field intensity in MATLAB. Other parameters (i.e., the resolution and transmission power) could be controlled by the console.

In the simulations, the wave intensity *I* was calculated as

4 $$I=\frac{{p}^{2}}{\rho c}.$$

**Figure 7:**
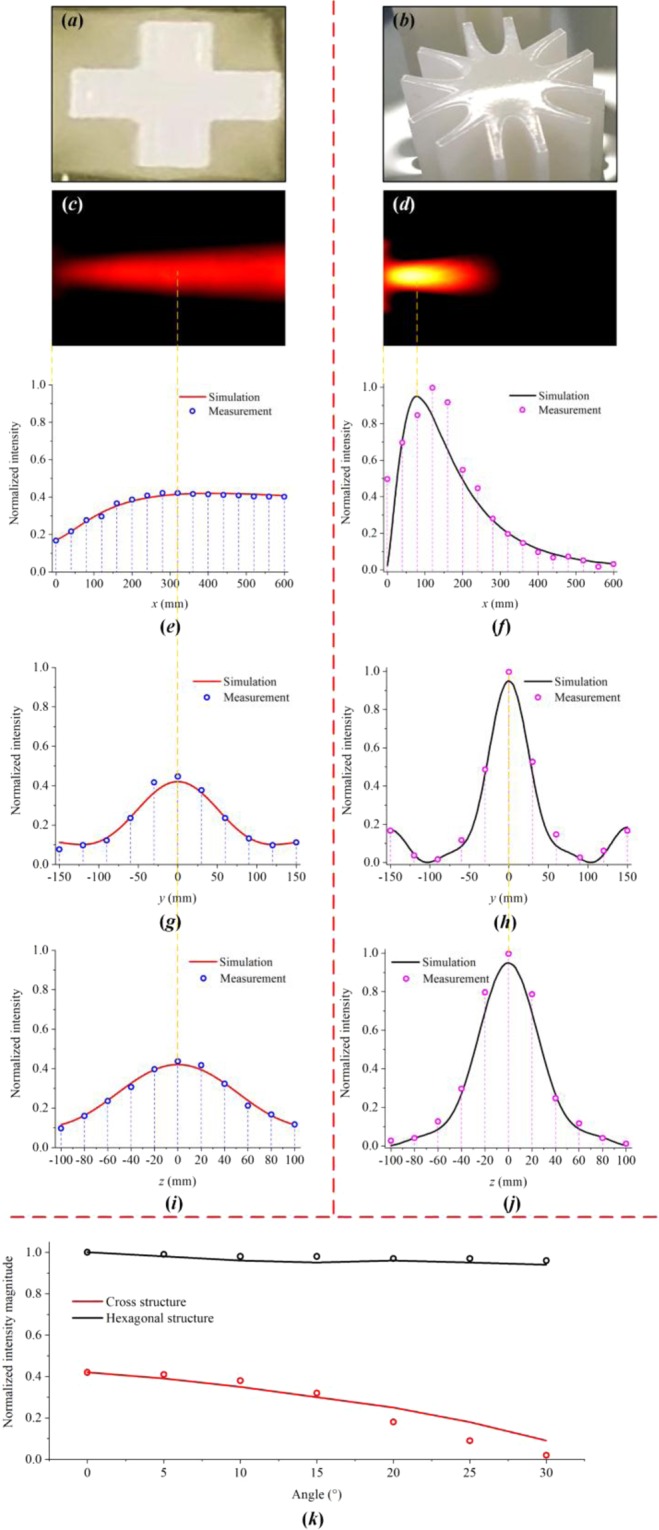
Simulated and experimental results and wave intensity distributions along each axis: (**a**,**b**) Unit cell shapes for the CS- and CH-structured lenses used in the experiments. (**c**,**d**) Experimental images. (**e**,**j**) Normalized wave intensity distributions along the *x-*, *y-* and *z*-directions. (**k**) Wave intensities at the focal positions with different incidence angles and unit cell structures.

By contrast, the wave intensity in the experiments could not be directly obtained because the probes (piezoelectric ultrasonic sensors) recorded data in the form of obtains voltage values rather than the acoustic field intensity itself. Therefore, to avoid signal conversion errors and facilitate comparative analysis, we normalized the simulated and experimental results to ensure that their values would always be consistent (Fig. 7). The measurement procedure was implemented by sweeping all preset data acquisition positions: 16 scans in the *x*-direction and 11 scans in the *y-* and *z*-directions. Thus, the entire distribution of the acoustic field intensity could be visualized after data processing and image smoothing (Fig. 7(c, d)). From the results, it can be intuitively observed that the two structures exhibit great differences in their focusing capabilities, including focusing intensity and focal length. Figure 7(e,j) show the normalized intensity distributions along the *x-*, *y-* and *z*-directions from the simulations and experiments, where the acoustic field distributions on each axis, including for the CS structure, are normalized with respect to the maximum value for the CH structure. Comparisons between Fig. 7(e,g,i) and Fig. 7(f,h,j) show that both the CS and CH structures achieve good focusing performance. More importantly, the composite lens with the CH structure exhibits excellent focusing characteristics while maintaining the 3-D focusing capability of a traditional composite lens. The consistency between the simulated and experimental results demonstrates that the lens with the CH structure behaves consistently with the theoretical analysis, thus proving the feasibility of underwater wave focusing with the newly designed CH structure.

To verify the multidirectional focusing capabilities of the composite lenses with different structures, an additional experiment was performed (Fig. 7(k)). To facilitate comparison between the simulated and experimental results, the CS structure used here was not concave (see Fig. 7(a)). Figure 7(k) shows the normalized intensity magnitudes at the focal positions for different incidence angles and unit cell structures. The wave intensities remain almost unchanged as the incidence angle varies for the lens with the CH structure. However, for the lens with the CS structure, the intensity of the wave is substantially attenuated as the angle increases. A rapid decrease in wave intensity is evident in the experimental results when the incidence angle is larger than 20°, which is due to the limitations of the pool boundaries.

## Conclusions

In conclusion, a new convergent acoustic lens with a CH structure is proposed in this paper; this lens can achieve a high focusing intensity and multidirectional focusing in an underwater system. Compared with traditional CS-structured composite lens, the newly designed lens has a wave focusing intensity that is more than twice as high and maintains the capability of 3-D focusing. In Particular, a composite lens with the CH structure can focus acoustic waves over a wide range of incidence angles without losing its focusing intensity. Moreover, the experimental results are consistent with simulations, thus validating the underlying theory of the new composite acoustic lens design.

## Data Availability

No additional data (other than those presented in the manuscript) were produced or used for the preparation of the manuscript.
